# Inherent Flame-Retardant, Humid Environment Stable and Blue Luminescent Polyamide Elastomer Regulated by Siloxane Moiety

**DOI:** 10.3390/polym14091919

**Published:** 2022-05-09

**Authors:** Qianqian Qi, Zhe Xiao, Yaowei Wang, Xinjin Yan, Peng Fu, Xiaomeng Zhang, Wei Zhao, Xinchang Pang, Minying Liu, Qingxiang Zhao, Zhe Cui

**Affiliations:** Henan Key Laboratory of Advanced Nylon Materials and Application, School of Materials Science and Engineering, Zhengzhou University, Zhengzhou 450001, China; qqbaozi@126.com (Q.Q.); xiaozhe15537119823@163.com (Z.X.); wangyaoweiwin@163.com (Y.W.); Yanxj0213@163.com (X.Y.); fupeng@zzu.edu.cn (P.F.); zhangxm@zzu.edu.cn (X.Z.); zhaoweizhao11@163.com (W.Z.); pangxinchang1980@163.com (X.P.); lmy@zzu.edu.cn (M.L.); zhaoqingxiang1@126.com (Q.Z.)

**Keywords:** polyamide copolymer, one-pot melt polycondensation, flame retardancy, hydrophobic character, blue luminescence

## Abstract

The rapid development of the polymeric materials market has created an urgent demand for the thermoplastic polyamide elastomer (TPAE) owing to its greater functionality, and ability to be synthesized via a facile and industrial route. In this work, a series of novel silicone-containing polyamides (PA1212/Si12) were successfully synthesized from 1,12-dodecarboxylic acid (LA), 1,12-dodecarbondiamine (DMDA), and 1,3-bis (amino-propyl) tetramethyldisiloxane (BATS), via a one-pot melt polycondensation method in the absence of a catalyst. FTIR, ^1^H-NMR, GPC and inherent viscosity results cohesively prove that the polymerization of monomers was well conducted, and the chemical structure was in high accordance with the design. As expected, the Si12 unit-content of the copolymers regulate the properties of the series. As the feeding ratio of BATS in the diamines increases from 5 mol% to 40 mol%, the thermal transition temperatures, *T*_g_ and *T*_m_, decline steadily before finally stabilizing at ~6 °C and 160 °C, respectively, indicating that the co-polyamides possess improved chain flexibility but restricted crystallization ability. The conspicuous evolution in crystalline morphology of the series was observed by XRD and AFM. The increased PA Si12 phase induces the crystallized PA 1212 phase to transit from a thermally-favorable large and rigid crystal structure (α phase) to a kinetically-favorable small and ductile crystal structure (γ phase). Reflected in their stress–strain behavior, PA1212/Si12 copolymers are successfully tailored from rigid plastic to ductile elastomer. The tensile strength mildly drops from above 40 MPa to ~30 MPa while the reversible elongation increases from ~50% to approximately 350%. Accordingly, the moderate surface tension differences in the monomers facilitate the efficient conduction of the co-polymerization process, and the distributed short siloxane unit in the backbone fulfills the copolymer with desirable elasticity. Interestingly, the novel silicone-containing polyamides also display Si12 unit-content dependent flame retardancy, humidity stability, and unconventional solid-state fluorescence properties. The elastomers exhibit a low bibulous rate and anti-fouling characteristics to dye droplets and mud contamination, pass the V–1 rating (UL 94) with a constantly declining PHRR value, and emit blue luminescence under a 365 nm light source. Herein, we propose a new facile strategy for developing a high-performance and multifunctional silicone-modified polyamide, which bears promising industrialization potential. In addition, this first reported silicone-containing thermoplastic polyamide elastomer, which is self-extinguishing, anti-fouling and blue-luminescent, will further broaden the application potential of thermoplastic polyamide elastomers.

## 1. Introduction

Thermoplastic polyamide elastomers (TPAEs) have emerged as important thermoplastic elastomers (TPEs) with significant potential. In addition to being a lightweight material, they bear comprehensive mechanical properties, excellent abrasive resistance, efficient energy return and an advantageous flexural efficiency over a wide temperature range [1,2]. As the latest member of the TPE family, TPAEs continue to attract ever-growing interest owing to their substantial application in diverse areas such as high-end sporting goods, medical instrumentation, automotives, transportation, telecommunication industries and even novel electron devices [3,4,5,6].

The typical chemical structure of TPAE is defined as a segmented copolymer composed of a polyamide (PA) hard segment (HS) and polyether or polyester soft segment (SS) [7,8,9], in which polyether represents the most popular soft segment choice. In addition, the mechanical performance of the TPAEs can be tailored from hard and rigid to soft and flexible by manipulating the molecular weight, type or the relative content of the two segments [3]. As recognized, the soft segment is mainly responsible for TPAE’s functionality, and the matrix stability is mainly in charge of the PA segment by constructing the intra- and interchain hydrogen bonds [10,11]. As the predominant TPAE species, the polyether segment in the poly(ether-co-amide) elastomer additionally gives the copolymer a permanent antistatic character and outstanding CO_2_ separation performance, which expands its application to films/gas-separation membranes and permanent antistatic agents. However, the hydrophilicity of the polyether results in higher water uptake and less dimensional stability. Notably, the flammability of the TPAE species represents a significant concern. Unfortunately, the physical–mechanical properties are excessively emphasized in current TPAEs [1]. To date, the functionality of existing TPAEs remains relatively limited, especially when compared with other TPEs [1]. Consequently, this has heavily restricted its expansion in potential areas of application, including microelectronics, precision-based products, and as a flame-retardant. Thus, there is an urgent demand for both TPAEs with greater functionality and rapid development of the polymeric materials market [1].

Organic-silicon polymers, composed of a Si-O backbone with two organic groups attached to each silicon atom, represent a popular material in biomedical, aerospace, automobile, and electric industries [12]. The high bond energy (460 kJ/mol) and long bond length (1.622 Å) of the Si-O bond and the organic/inorganic nature of the main chain endow the polymer with extremely high chain flexibility (*T*_g_ around −120 °C), good thermal and oxidative stability, low surface tension, high gas permeability, excellent dielectric properties and physiological inertness or biocompatibility [12,13]. Furthermore, the latest research illustrates that the introduction of organic-silicon components in the polymer also enhance its flame-retardant properties due to the formation of a SiO_2_ barrier layer during the combustion process [14,15,16]. In direct relation to the interesting combination of the unique chemico-physical properties offered by the siloxane structure, silicone-containing copolymers continue to receive interest from academics and various industries.

Indeed, the siloxane structure has been employed as a modifying component for thermoplastic polyurethane elastomers (TPUs) [17,18], and thermoplastic polyurea elastomers (TPUys) [19,20], achieving great success in the commercial industry. However, the research of silicone-modified polyamide (PAS) has progressed rather slowly. Kajiyama et al. pioneered the synthesis of a class of polydimethylsiloxane(PDMS)-aramid multiblock copolymers ranging widely in composition via low-temperature solution polycondensation [21,22]. The inherent viscosities of the synthesized copolymers all measured below 0.8 dL/g, and decreased sharply with increased PDMS content. In addition, the new copolymers exhibited a typical rigid plastic nature with an abrupt break at an elongation less than 50% even with a PDMS content of almost 70%. Through low-temperature solution polycondensation, Akashi et al. synthesized a series of PAS resin based on aramid and PDMS or BATS [23,24,25,26,27,28,29,30]. Similarly, this series possessed low inherent viscosity and elongation strain. Moreover, their research focused on the exploration of surface properties and gas permeability aiming at biomedical applications. Rached et al. developed a series of PA12-*b*-PDMS-*b*-PA12 triblock copolymers through the anionic ring-opening polymerization of lactams with α, ω-dicarbamoyloxy caprolactam PDMS obtained by the reaction of isocyanates with silanol groups as the macroinitiator [31,32]. The synthesized triblock copolymer was expected to enhance the mechanical properties of PA12/PDMS when used as a compatibilizer. Poojarl et al. synthesized PAS via ammonia-propyl-terminated PDMS and terephthalic acid using lipase-catalyzed methodology [33]. They ultimately obtained the glue-like but highly viscous and sticky product although its molecular weight reached 60,000 g/mol, rendering its use impractical. PA6 and PA1212 are the two main reported aliphatic HS for PAS design. Li et al. reported the synthesis of PDMS-PA6 multiblock copolymers by bulk polymerization [34]. Further investigation focused on their thermal properties and application as a plasticizer for PA/SiO_2_ composite. A series of PA1212-*b*-PDMS with stable mechanical properties and thermal stability were reported in our previous work via a prepolymer polycondensation route [35]. Excellent biocompatibility and interface properties indicated their broad application prospects in the biomedical field. Unfortunately, although the copolymer possessed improved elongation at break, the stress–strain curve of the series still displayed the basic characteristics of ductile plastics. To our knowledge, there have been no Si-O structure-containing TPAE species reported to date. The severe phase separation of siloxane containing moieties from other components both in the reaction and copolymer bulk may represent the main challenge in achieving a silicone-modified thermoplastic polyamide elastomer with robust mechanical properties via a facile and industrial synthesis route.

Herein, we propose a new strategy to advance the study of silicon-containing polyamide copolymers. A one-pot polycondensation method is selected to prepare the high-performance poly (silicone-*co*-amide) copolymer (PA1212/Si12 copolymers) by employing 1,12-dodecarboxylic acid (LA), 1,12-dodecarbondiamine (DMDA) and 1,3-bis(amino-propyl) tetramethyldisiloxane (BATS) monomers directly. The shortest Si-O-Si structure siloxane, BATS, is chosen to minimize the polarity difference in both reactants and resulting copolymer bulk. In addition, the one-pot polycondensation method is employed to help effectively distribute the BATS unit in the main chain of the copolymers, which may improve compatibility between the aliphatic amide unit and BATS unit.

Through precisely controlled synthesis, a series of PA1212/Si12 copolymers with a 5–40% feeding ratio of BATS in diamides are designed and prepared. The chemical structure of the novel series was determined by FT-IR and ^1^H NMR. Furthermore, the thermal transition, crystallization and mechanical behavior of the novel series were investigated. Their performance in combustion, humid-environment and fluorescence were further explored. Conclusively, the first multifunctional silicone-containing TPAE species is successfully designed and synthesized, broadening the application potential of the TPAE family to more fields.

## 2. Materials and Methods

### 2.1. Materials and Agents

The 1,12-dodecarboxylic acid (LA) was purchased from Zibo Guangtong Chemical Co., Ltd. (Zibo, China) and 1,12-Dodecarbondiamine (DMDA) was purchased from Henan Junheng Group Co., Ltd. (Puyang, China). Both are industrial grade and used as received. The 1,3-bis (amino-propyl) tetramethyldisiloxane (BATS) was purchased from Meryer (Shanghai) Chemical Technology Co., Ltd. (Shanghai, China). Hexafluoroisopropanol (HFIP), metacresol, absolute ethyl alcohol, trichloromethane, trifluoroacetic-D and methanol-D, trifluoroacetic-D and methanol-D were purchased from Shanghai Aladdin BioChem Technology Co., Ltd. (Shanghai, China). All compounds are of analytical purity and used as received.

### 2.2. Synthesis of PA1212/Si12 Copolymers

**Figure 1:**
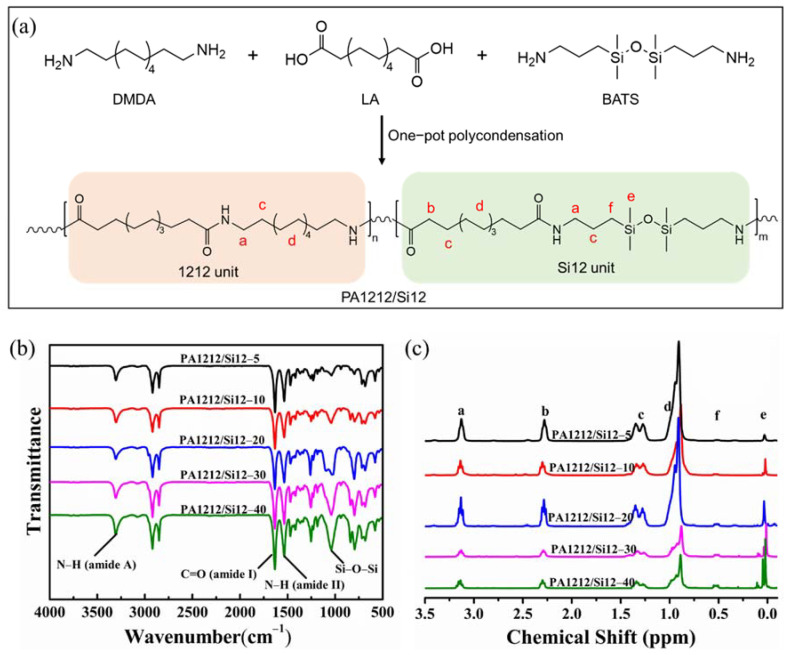
(**a**) One–pot polycondensation route of PA1212/Si12 copolymers; (**b**) FTIR and (**c**) ^1^H NMR spectra of PA1212/Si12 copolymers.

PA1212/Si12 copolymers were synthesized via one-pot melt polycondensation route as illustrated in Figure 1a. The typical procedure is described as follows: Predetermined amounts of LA, DMDA and BATS were charged in a 200 mL stainless steel reactor, first. Next, the reactor was purged three times with nitrogen to ensure the following reaction was carried out under inert atmosphere. After that, the reaction system was slowly heated while stirring. The whole polycondensation followed this procedure: slowly heat to 230 °C, hold for 4 h, then steam release to atmospheric pressure and vacuumize to ∼90 Pa for 1 h. The pressure in the first two stages was kept below 0.5 MPa, and the mole feeding ratio of BATS in the diamine quantity was adjusted in the range of 5% and 40%. Finally, a series of PA1212/Si12 copolymers with various Si12 unit content were achieved, which were labelled as PA1212/Si12-(5, 10, 20, 30, 40), respectively, for brevity. The number corresponds to the mole feeding ratio of BATS.

Prior to characterization, all products were extracted with ethanol for 36 h to remove the residual monomer and vacuum dried at 80 °C.

### 2.3. Characterization

All films used for the relevant tests were unified and prepared on a HT500 hot press (Xutaiheng, Shenzhen, China), and the preparation process is as follows: A 0.5 g quantity of purified and dried polymer particles were placed in the middle of two PTFE films and placed on the hot press stable. With 2 MPa pressure, the molding part was heated to 190 °C, and kept for 3 min. Then, after naturally cooling the prepared sample was extracted. The thickness of the prepared films was controlled to around 1 mm.

All strips used for the relevant tests were prepared on a SZS-20 microinjection machine (Ruiming, Wuhan, China) based on same process with different molds. The process route is as follows: Fill the particles in the container, hold for 5 min then start to work with a 0.3 MPa injection pressure and 0.4 MPa holding pressure, respectively.

All samples used for the relevant tests were dried in vacuum oven for 24 h at 80 °C before testing.

Fourier transform infrared spectroscopy (FTIR) was conducted on a TENSOR-Ⅱ spectrometer (Bruker, Karlsruhe, Germany) with films under ATR mode. The spectra were recorded from 4000 cm^−1^ to 500 cm^−1^ with a spectral resolution of 4 cm^−1^ and the signals were averaged over 64 scans and normalized to eliminate the differences in film thicknesses.

The ^1^H NMR spectra were recorded by a DPX400 spectrometer (Bruker, Karlsruhe, Germany) at ambient temperature. CD_3_OD was used as the solvent for BATS, D_2_O for DMDA and CF_3_COOD for LA and PA1212/Si12 copolymers. All the deuterium reagents do not contain tetramethylsilane.

The relative viscosity (*η_r_*) of the polymers was tested via Ubbelohde viscometer (HAD 1835) method. The reference solvent comprised a mixture of chloroform and metacresol with a volume ratio of 2:3, and the solute content was 5 mg/mL. The relative viscosity *η_r_* was calculated by Equation (1), where *t* represents the flow time of the polymer solution and *t_0_* represents the flow time of the reference solvent. Then, the inherent viscosity ([*η*]) was calculated by Solomon–Ciuta Equation (2), where *η_sp_* = *η_r_* − 1, *c* is the concentration of the silicone-modified polyamide.

(1) $$\eta_{r}=\frac{t}{t_{0}}$$

(2) $$\left[\eta \right]=\sqrt{2\left(\eta_{sp}-ln\eta_{r}\right)}/c$$

Gel permeation chromatographic (GPC) experiments were performed on Waters GPC1515 instrument equipped with a refractive index detector at room temperature. HFIP containing sodium trifluoroacetate (0.005 mol/L) was used as the eluent at a rate of 1 mL/min and PMMA was used as the calibration standard.

Differential scanning calorimetry (DSC) was performed on DSC-Q8500 (PerKinElmer, Waltham, MA, USA) under N_2_ atmosphere. The glass temperature was measured with a rate of 100 °C/min while the melting temperature was measured with a rate of 10 °C/min.

WAXD data were recorded on Rigaku XRD Ultima IV X-ray diffractometer, with Cu Kα radiation (λ = 0.154 nm). Data were collected in the range of 2θ = 10~50° with a scanning speed of 5°/min. Radiation parameters were 40 kV and 20 mA.

Atomic force microscopy (AFM) was carried out on NanoWizard^®^4XP BioScience (Bruker, Karlsruhe, Germany) in non-contact mode. Moreover, 0.3 wt.% of sample solutions were prepared by dissolving the copolymers in HFIP. Thin films were obtained by spin coating the 0.3 wt.% solutions on a 1 cm × 1 cm silicon wafer at 3000 rpm/min for 1 min.

Tensile tests were carried out on dumbbell specimens (total length, 75 mm; width of parallel part, 5 mm; thickness, 4 mm) by a CMT5104 universal tester (MTS, Shenzhen, China) with a speed of 20 mm/min. The tensile strength and ultimate elongation were obtained from stress–strain curves.

Thermal gravimetric analysis (TGA) was conducted on a Q50 (TA, New Castle, DE, USA) by heating a sample from 50 °C to 600 °C at a rate of 10 °C min^−1^ under nitrogen atmosphere and air atmosphere, respectively. Moreover, 5–10 mg samples were placed in small aluminum pans for analysis. The corresponding weight loss curve was used to investigate the thermal stability of the samples.

Underwriter Laboratory 94 vertical burning (UL-94) and micro-cone calorimeter measurements were used to investigate the flame retardancy of PA1212/Si12 copolymers. UL-94 measurements were performed on CZF-2 equipment with a dimension size of 6.0 × 13 × 3.8 mm^3^. The micro-cone calorimeter tests were carried out using an FAA-PCFC micro-cone calorimeter with ~4 mg powder samples.

The water contact angle was determined by the static contact angle of water droplets on the flat copolymer films using DSA10-MK2 instrument (KRüSS GmbH, Hamburg, Germany). For one film, at least five positions were measured. Test conditions were as follows: the ambient temperature was 25 °C, the environment humidity was 65%, and the droplet size was 0.5 μL. The dye dripping test was performed under room temperature. A 0.05 g/mL quantity of Congo red water was dropped onto the film surface then held for 10 s, the sample was subsequently washed and dried. For the mud fouling test, film samples were buried in wet mud at 5 cm depth for a week. The surface fouling was then washed and dried.

Ultraviolet absorption spectra of all samples were recorded on a UV/VIS/NIR Spectrometer Lambda 1050+ (PerkinElmer, Waltham, MA, USA). The spectra were recorded from 200 nm to 500 nm. The samples were prepared as films of uniform thickness before testing.

The fluorescence excitation and emission tests of the films with a unified thickness of 0.1 mm were performed on a spectrometer at ambient temperature with a 365 nm excitation wavelength. The spectra were recorded from 400 nm to 700 nm.

## 3. Results and Discussion

### 3.1. Synthesis and Characterization of PA1212/Si12 Copolymers

As shown in Figure 1a, PA1212/Si12 copolymers were synthesized with LA, DMDA and BATS monomer directly via one–pot polycondensation route. Five kinds of PA1212/Si12 were synthesized with a different feeding ratio (5, 10, 20, 30 and 40 mol%) of BATS in the diamines. For brevity, PA1212/Si12 copolymers were named as PA1212/Si12-5, 10, 20, 30 and 40 corresponding to 5, 10, 20, 30 and 40 mol% feeding ratio of BATS, respectively. Before further characterization, the prepared copolymers were purified by ethanol extraction and dried at 80 °C under vacuum for 12 h. Detailed information of the extraction rate, named as residue rates of the monomer, is listed in Table 1.

FTIR was first performed on the prepared polymers and the monomers, as shown in Figure 1b and Figure S1a. All PA1212/Si12 copolymers exhibit similar spectra with the characteristic absorption peaks, including the peaks of amide group (amide A, Ι, II) and the peaks of BATS unit. Clear differences are observed among the spectra of the monomers and PA1212/Si12 copolymers. The wide absorption band at ∼3460 cm^−1^ attributed to the stretching vibration of O-H in LA disappears on the spectra of PA1212/Si12 copolymers and a new absorption band at ∼3302 cm^−1^ attributed to amide A (ν_N-H_) appears in the copolymers’ spectra. The characteristic absorption band at ~1579 cm^−1^ assigned to the N-H bending vibration of DMDA and BATS disappears after the polymerization while the carbonyl bands of amide I (C=O stretch) and amide II (N-H) raise to ~1640 cm^−1^ and ~1540 cm^−1^ in the PA1212/Si12 copolymers’ spectra, respectively. Moreover, the stretching vibrations of C-H (methylene segment), the bending deformation of C-H (Si-CH_3_), the stretching vibration of Si-O-Si and the stretching vibration of C-H (Si-CH_3_) are well located in PA1212/Si12 copolymers’ spectra, at 2930~2840, ~1261, 1043 and 800 cm^−1^ [36,37], respectively. All observed absorption bands in the spectra simultaneously indicate that the PA1212/Si12 copolymers were synthesized successfully based on the design. Furthermore, as shown in Figure 1b, the absorption peaks of BATS units appear to clearly be content dependent. The stretching vibration band of Si-O-Si at 1043 cm^−1^ becomes increasingly pronounced as the BATS feeding ratio increases. As illustrated in Figure 1b, the Si-O-Si stretching vibration band is solely contributed to by the Si12 unit, while the N-H stretching vibration is contributed to by all the amide units. Herein, the content of Si12 unit could be deduced by comparison of the two peaks’ areas, and the results are provided in Table 1.

The ^1^H NMR spectra of PA1212/Si12 copolymers and the monomers are shown in Figure 1c and Figure S1b. The signals at 0.86–0.91 ppm are attributed to the chemical shift of the methylene group (d) far from the carboxyl or amine group in LA and DMDA unit. The signals at 1.25–1.32 ppm are assigned to the chemical shift of β-C group (c) of all units. The signals at 2.27 ppm and 3.10 ppm are attributed to the chemical shift of α-C group of the LA unit (b), DMDA and BATS unit (a), respectively. The signals at 0 ppm and 0.56 ppm are ascribed to the chemical shift of Si-CH_3_ (e) and α-C in the methylene group (f) of BATS unit, respectively. These together manifest the successful preparation of PA1212/Si12 copolymers. The spectra of the five PA1212/Si12 copolymers are basically similar while the chemical shifts of (e) and (f) appear increasingly prominent with the growing content of the Si12 unit. As known, the intensity of the proton peak in the ^1^H NMR spectrum is quantitatively related to the number of protons in the chemical structure. Thus, the actual content of the Si12 unit in PA1212/Si12 copolymers could be calculated by the comparison between the peak area of Si-CH_3_ (e) and α-H of LA (b). The calculated results are provided in Table 1. As shown in Table 1, the actual content of Si12 unit calculated from ^1^H NMR ranges from 3.2% to 29.3%. which is very close to the FTIR calculated data. This not only proves the reliability of the two calculation methods, but also indicates the stability of the chemical structure of the copolymers. Compared with the theoretical ratio, a clear content drop is noticed. Interestingly, the gaps between the actual content and theoretical ones narrow as the feeding ratio increases, which may be ascribed to the higher volatility and lower reactivity of BATS than DMDA.

In order to further verify the rationality of the synthesis route, the relative molecular weight, intrinsic viscosity and monomer residue rates of PA1212/Si12 copolymers were performed by GPC, Ubbelohde viscometer and Soxhlet apparatus, respectively, and all results are collected in Table 1. As presented, monomer residue rates of PA1212/Si12 copolymers are relatively low, from 1.60 wt.% to 3.23 wt.%, indicating that the polycondensation of LA and BATS and DMDA monomers were well carried out with a high extent of reaction. All PA1212/Si12 copolymers bear a high intrinsic viscosity, climbing from 1.2 to 1.9 dL/g as the feeding ratio of BATS increases, just corresponding to the lower BATS loss in the high feeding ratio. The relative molecular weights of PA1212/Si12 copolymers are also at a high level. However, PA1212/Si12-40 does not show a corresponding relationship between the relative molecular weight and instinct viscosity, where both the number average molecular weight and weight average molecular weight are the lowest in the series of copolymers, 5.5 kg/mol and 13 kg/mol, respectively. The reason for this may be the greatest difference between PA1212/Si12-40 and the GPC reference compound, PMMA, in chain flexibility. Thus, as-prepared copolymers all possess high polymerization degree with a well-controlled molecular weight distribution (PDI < 2.4).

In general, high-molecular-weight PA1212/Si12 copolymers with a designed chemical structure are successfully obtained by the proposed one-pot polycondensation route. The route effectively solves the phase separation problem in the reaction between a silicone moiety and polyamide moiety and improves the reaction efficiency and structure design feasibility. Moreover, the one-pot route can be easily applied to mass production due to its simple operation.

### 3.2. Thermal Transition and Crystallization Behaviors

**Figure 2:**
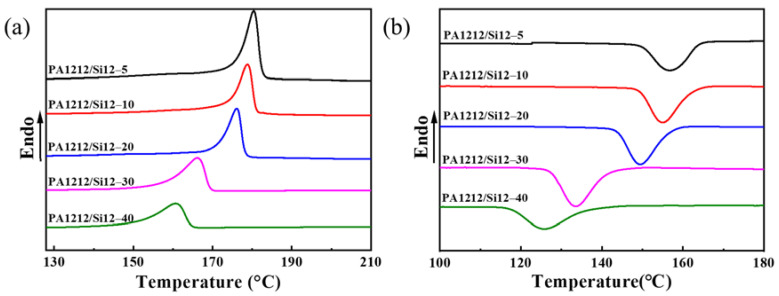
DSC curves of PA1212/Si12 copolymers during (**a**) the first cooling scan and (**b**) the second heating scan at 10 °C/min.

The thermal transition temperatures of samples were determined by DSC, as presented in Figure 2, Figures S2 and S3. The corresponding parameters are collected in Table 2. As shown, the glass transition temperature (*T*_g_), melting temperature (*T*_m_), and crystallization temperature (*T*_c_) of the series, are all lower than those of the PA1212 homopolymer shown in Table S1, and present a continuous downward trend as Si12 unit-content increases. Specifically, from PA1212/Si12-5 to PA1212/Si12-40, *T*_g_, *T*_m_ and *T*_c_ decrease from 30 °C to 6 °C, 179 °C to 161 °C, and 157 °C to 126 °C, respectively.

Glass transition represents a relaxation phenomenon of the amorphous phase of the polymer or amorphous polymer from the frozen state to thawing state. In addition, *T*_g_ is defined as the temperature at which chain segments in polymer start to move. Generally, the lower the *T*_g_, the better the mobility of the chain. Thus, the constantly decreasing *T*_g_ value indicates the continuously improving mobility of PA1212/Si12 copolymer chain as the Si12 unit-content also increases. Interestingly, PA1212/Si12-20 seems to be a critical point, which will be evidenced again in the following discussion. Furthermore, the chain transitions from a glassy state to a highly elastic state at room temperature with a *T*_g_ value approaching 0 °C. It is foreseeable that this drastic change in chain mobility will inevitably enact some desirable evolution of performance, especially in mechanical properties, which is precisely what we expect.

Melting and crystallization are very complex processes. As known, *T*_m_, is regulated by both thermodynamic and kinetic factors, such as crystal form, crystal grain size, crystal integrity, thermal history, and so forth. Regarding the copolymer, the sequence structure of the chain significantly affects its *T*_m_ value. Compared with the corresponding homopolymer, a mild decrease in *T*_m_ value is common for the segmented copolymers, and a monotonic decrease until reaching a eutectic point in *T*_m_ value is typical for the random copolymers [38,39]. In our study, only one exothermal peak is observed in the heating curve of PA1212/Si12 copolymers from −120 °C to 210 °C. It is reasonable to attribute it to the melting of PA1212 domain in the copolymers, since the silicone domain possesses a much lower *T*_m_ value [35]. Moreover, the variation trend of *T*_m_ for the series indicates that the random copolymerization is more likely to occur through our one-pot polycondensation route. Consistent with the variation in *T*_g_, when the content of Si12 unit is lower than 10% (PA1212/Si12-5 and PA1212/Si12-10), *T*_m_ values are close to PA1212 homopolymer. Then, as the Si12 unit-content continues to climb, the *T*_m_ value decreases sharply. With regard to the crystallization process, nucleation and crystal growth represent the two dominating factors. In general, nucleation is the main controlling factor for high-temperature crystallization, and crystal growth (chain mobility) mainly controls low-temperature crystallization. Thus, the illustrated increasing chain mobility from PA1212/Si12-5 to PA1212/Si12-40 effectively explains the continuous decrease of the *T*_c_ value. By comparing the difference between *T*_m_ and *T*_c_, the degree of undercooling (Δ*T*) can be deduced. A continuously decreasing trend is also found, while compared with the PA1212 homopolymer, the variation in Δ*T* seems interesting, which initially drops slightly and then raises. As known, Δ*T* represents the crystallization ability to some extent [40,41]. The lower value of Δ*T* indicates a faster crystallization rate or higher crystallization tendency [42]. Compared with homopolymer, the initial relative lower and then higher Δ*T* value implies that the introduction of BATS unit initially improves the crystallization ability, and then the crystallization retracts as the amount increases.

Thus far, we can observe a more comprehensive perspective regarding the melting and crystallization of the series. The introduction of the flexible BATS unit with methylene side groups and longer covalent bonds greatly improves the mobility of the polymer chain. However, the insertion of BATS unit also destroys the regularity of the original chain. Moreover, the steric hindrance effect from the side groups of BATS unit limits the effective proximity between chains, which in turn adversely affects the formation of the interchain and intrachain hydrogen bonds. The improvement of chain mobility assists crystal growth, while the decrease of chain regularity and the weakening of hydrogen bonding jointly restrict the crystallization. As illustrated, in this series, the thermodynamic factors overwhelm the kinetic factors. Consequently, the transition temperatures corresponding to the melting and crystallization processes decrease along with the Si12 unit-content. In terms of the crystallization speed or tendency, when the feeding ratio of BATS is not excessively large, a slight improvement is observed. Reflected in the crystallinity of the PA1212 domain (*X*_c_), a slight increase followed by a sharp decrease is identified, and the turning point appears again at PA1212/Si12-20. However, it still retains a relative high crystallinity (~35%) even for PA1212/Si12-40, which benefits from the strong crystallization ability of PA1212. In addition, maintenance of the semicrystalline nature of the PA1212 domain will provide a foundation for stable elastomer performance. Notably, PA1212/Si12-20 is a critical sample, and all parameters display a sudden change at this content.

**Table 2:** The thermal transition parameters of PA1212/Si12 copolymers.

Sample	*T*_g_ (°C)	*T*_m_ (°C)	*T*_c_ (°C)	Δ*T* (°C)	Δ*H* (J/g)	*X*_c_^a^ (%)
PA1212/Si12–5	30	179	157	22	52.0	44.0
PA1212/Si12–10	21	178	154	24	51.7	45.2
PA1212/Si12–20	19	174	149	25	45.4	43.2
PA1212/Si12–30	5.8	165	134	31	32.3	33.2
PA1212/Si12–40	5.7	161	126	35	30.4	35.4

^a^ Crystallinity of PA1212 domain in the copolymers and the standard enthalpy is 121.8 J/g [43].

**Figure 3:**
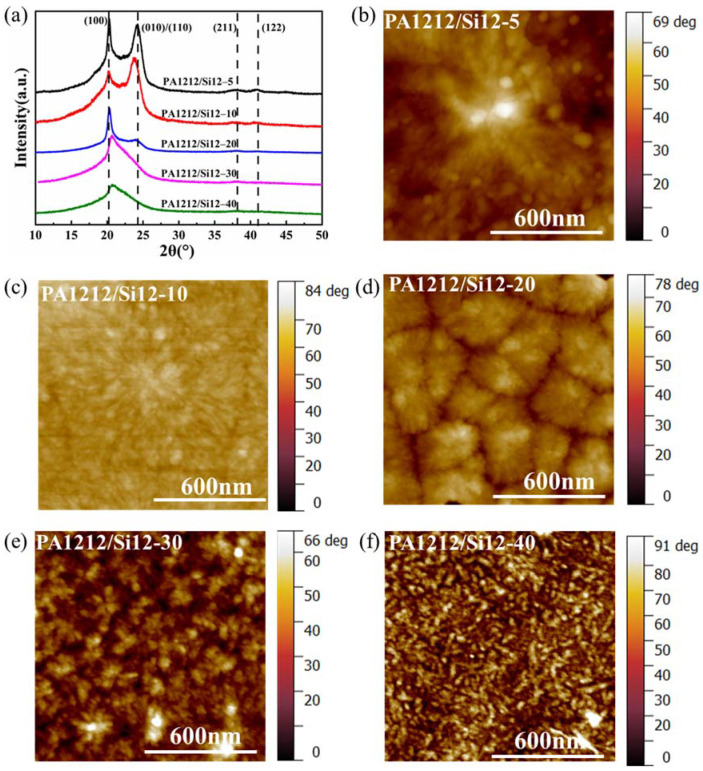
The WAXD patterns (**a**) and the AFM phase images (**b**–**f**) of PA1212/Si12 copolymers.

WAXD and AFM were utilized to further explore the phase morphology of the series regulated by Si12 unit. As shown in Figure 3a, the WAXD patterns of the five copolymers exhibit clear differences. The diffraction peaks and intensities vary with the content of Si12 unit. Furthermore, when the content of Si12 unit in the copolymer is low (PA1212/Si12-5, -10), the diffraction pattern is characterized by diffraction peaks at 2θ = 20.3°, 24.1°, 38.1° and 40.8°, which is identical with the triclinic α-crystal form of PA1212 (Figure S4) [44,45]. The diffraction peaks at 20.3° and 24.17° are attributed to (100) crystal plane and (010)/(110) crystal plane, respectively. The diffraction peak at 38.2° and 40.9° are attributed to (211) crystal plane and (122) crystal plane, respectively. Generally, for the aliphatic polyamide, the *d*_spacing_ of (100) crystal plane reflects the chain spacing within the hydrogen bonding sheets, the *d*_spacing_ of (010)/(110) crystal plane reflects the spacing between the hydrogen-bonded sheets, and the (211) and (122) crystal planes are generated by the stacking of the methylene groups. As the Si12 unit-content rises (PA1212/Si12-20 to PA1212/Si12-40), the diffraction peaks of (211) and (122) crystal planes become inconspicuous promptly. This may result from the better flexibility of the Si12 unit and the existence of four side groups in the structure, which hinders the ordered arrangement of methylene in the copolymer. Moreover, as the Si12 unit-content increases, the diffraction peak of the (010)/(110) crystal plane transforms from separated to dispersed, and finally merges with the diffraction of (100) crystal plane at around 2θ = 20.5, which is the typical diffraction pattern of PA1212 in the pseudohexagonal γ-crystal form [46]. As known, hydrogen bonds play a crucial role in polyamide materials. To reveal differences in the hydrogen bonding of the series, the hydrogen bonding index (HBI) is introduced, which is the quantity ratio of the hydrogen-bonded amide groups to the free amide groups [47]. The higher the HBI value, the higher the degree of hydrogen bonding in the copolymer. In polyamide, the stretching vibration of C=O is sensitive to its hydrogen bonding state. Reflected in the IR spectrum, the stretching vibration band of C=O (amide I, 1670~1580 cm^−1^) is contributed to by three factors: the stretching vibration of free C=O (around 1645 cm^−1^), the stretching vibration of disorder hydrogen-bonded C=O (around 1634 cm^−1^), and the stretching vibration of order hydrogen-bonded C=O (around 1623 cm^−1^). Due to the equal absorption coefficients, HBI can be calculated directly by comparing the peak area of the hydrogen-bonded C=O (ordered and disordered) to that of the free C=O. The deconvolution results of amide I and the calculated HBI are presented in Figure S5 and Table S2. As shown, the HBI values gradually decrease, as the Si12 unit increases. This indicates that the degree of hydrogen bonding is gradually weakened, resulting in the reduction of crystallinity and transition of the crystal form. The essentially unchanged (100) diffraction peak indicates that the formation tendency of hydrogen bonding among chains in the copolymers are still strong even when the content of Si12 unit reaches 29 mol% for PA1212/Si12-40 due to the high cohesive energy of the hydrogen bonding formed between the amide groups. While the intensity constantly decreases, gradually widening in the full width at half maximum (FWHM) of the diffraction peaks, implying decreases of the grain size and the deterioration of the crystal integrity. The crystal form of the copolymer becomes increasingly unstable, which is also evidenced by AFM below. As (010)/(110) crystal plane is constructed by Van de Waals, it is easy to understand that the adverse influence of the introduction of BATS unit on it is more severe compared with (100) crystal plane. Clearly, the content of Si12 unit in the copolymer regulates the crystal phase in grain size, crystal integrity and crystal form. Finally, the crystal phase converts from the thermodynamically-favorable rigid α-form domain to the dynamic kinetically-favorable ductile γ-form domain.

The AFM images of PA1212/Si12 copolymers are in good agreement with the WAXD patterns, as shown in Figure 3b–f. The images of PA1212/Si12-5 and PA1212/Si12-10 show considerable large, complete and closely arranged spherulite phase, corresponding to the typical crystal morphology of α-crystal form of PA1212 [48]. The crystal size of PA1212/Si12-20 is relatively uniform but small, and no complete spherulites can be observed. Moreover, two phases, PA1212 domain and PASi12 domain begin to appear in the image. For PA1212/Si12-30 and PA1212/Si12-40, the morphology is quite different. The fine crystalline phase uniformly distributes in the observation zone. The higher the Si12 unit-content, the finer the crystalline domain will be. Two phases are more clearly observed in the images with intermingled phase boundaries. Combined with the WAXD results, the fine crystal morphology should correspond to the γ-crystal form of PA1212. Generally, with the increasing content of Si12 unit, the crystal becomes more incomplete and the grain size decreases, which is consistent with the DSC and XRD results. Distinct from other PAS, no clear microphase separate phenomena are observed [27,49]. This indicates that the PASi12 domain and PA1212 domain exhibit good compatibility in PA1212/Si12 copolymers obtained by the proposed one-pot condensation route, which will be conducive to perform synergistically.

### 3.3. Mechanical Properties

**Figure 4:**
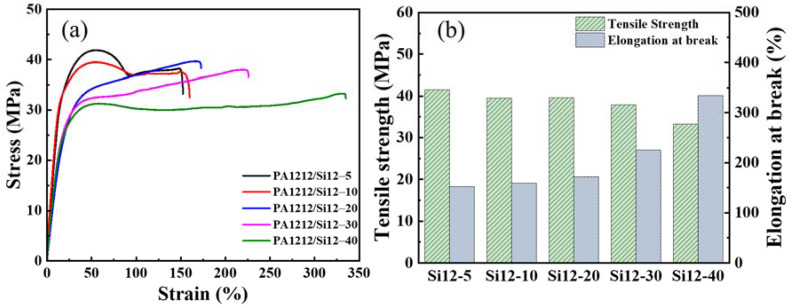
The typical stress–strain curves (**a**) and the tensile strength and elongation at break (**b**) of PA1212/Si12 copolymers.

The mechanical properties of PA1212/Si12 samples were measured by a tensile test at a rate of 10 mm/min at room temperature. The results are shown in Figure 4. PA1212/Si12-5 and PA1212/Si12-10 present a similar stress–strain curve with PA1212-*co*-PDMS segmented copolymers reported in our previous work [35], a typical curve of the plastics with an evident yield point at around 50% elongation. Excitingly, the distinct stress–strain curve, a typical stress–strain curve of TPE, is presented by PA1212/Si12-20, -30 and -40 with an elongation at break of 172%, 225%, and 334%, respectively. Finally, the copolymer is successfully transformed from a plastic to an elastomer by adjusting the Si12 unit-content. In addition, the critical sample is PA1212/Si12-20, just corresponding to the transformation of the crystal form from α phase to γ phase. For traditional TPAEs, the hard segment, crystalline polyamides, works as a physical crosslinking point while the soft segment, polyester, or polyether, provides elasticity. Thus, in PA1212/Si12 copolymer, the crystalline portion with fine ductile grains acts as the physical crosslinking point to ensure stability, while the amorphous portion containing flexible chain structures provides elasticity.

The tensile strength and the breaking strain of PA1212/Si12 copolymers all vary to different degrees, but the overall trend shows that the tensile strength decreases from 41.6 MPa to 33.4 MPa while the breaking strain increases from 152% to 335%. As manifested by DSC, XRD and AFM results, all PA1212/Si12 copolymers are semi-crystalline polymers and share a tendency by which *X*_c_ and the grain size decrease with increases in the content of Si12 unit. Moreover, as known, *X*_c_ and the grain size are positively correlated to the tensile strength, thus the tensile strength decreases correspondingly. The tendency of the strain reveals just the opposite due to the polymer chain becoming more flexible with the increasing content of Si12 unit, thus allowing motion rearrangement and other movements to be carried out with greater ease.

### 3.4. Thermal Stability

Thermal stability is one of the important indexes for evaluating the materials, especially their physical and processing properties. It is foreseeable that the copolymers possess greater thermal stability, since the bond energy of the Si-O bond (460 kJ/mol) is higher than that of the C-C bond (357 kJ/mol) [12]. Both TGA and DTG under N_2_ and air atmosphere conditions prove this, as shown in Figure S6 and Table 3.

The decomposition process of PA1212/Si12 copolymers is divided into two stages (the rapid stage below 500 °C and slow stage above 500 °C). By comparing with PA1212 homopolymer (see Table S3), it is not difficult to attribute the second stages to the contribution of BATS unit. Clearly, the thermal decomposition is delayed by the introduction of BATS unit, regardless of whether under a N_2_ or air atmosphere. Although the fluctuation exists among the series, the decomposition temperatures (*T*_5%_, *T*_max1_ and *T*_max2_) all show an apparent increase of 10 to 30 °C, compared with PA1212 homopolymer. In addition, when the reference species changes to the prepared PA1212-*b*-polyether (TPAE-22, homemade via reported literature [4]), the improvement is more significant, as illustrated in Table S3. Predictably, the much better thermal stability will endow the new series with greater application prospects.

Although the thermal decomposition temperatures obtained under air atmosphere are relatively lower than that under N_2_ atmosphere, the residues of PA1212/Si12 copolymers detected under air atmosphere are higher, varying from 2.21 to 4.75% with the increasing content of the Si12 unit. The residue is further analyzed by FT-IR spectrum (Figure S7). A typical SiO_2_·nH_2_O spectra is obtained, which is characterized by a wide absorption band of Si-O stretching vibration near 1061 cm^−1^ and the water absorption peaks at 3451 and 1634 cm^−1^. It can be seen that under air atmosphere, PA1212/Si12 will thermally decompose into silica, which has been reported as crucial for the flame retardation of silicone-containing materials. In addition, the residues of PA1212 homopolymer and the homemade TPAE-22 under air atmosphere are much lower than that of PA1212/Si12 copolymers. Therefore, considering both the residue rate and the decomposition product, the flame retardancy of the new series is expected to be greatly improved, which will be discussed in detail below.

### 3.5. Combustion Performances

As discussed above, the flammability of PA1212/Si12 copolymers are expected to be improved owing to the introduction of Si12 unit, which is more thermally stable and decomposes into silica under air atmosphere. For purposes of identification, a vertical burning test (UL-94) and micro-cone calorimeter test were performed.

**Figure 5:**
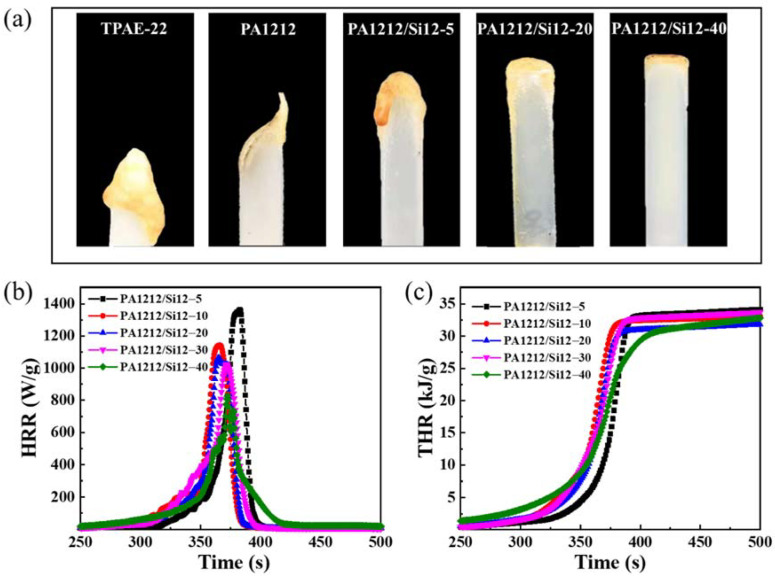
(**a**) Digital photographs of the samples after combustion; (**b**) HRR and (**c**) THR curves of PA1212/Si12 copolymers.

Polyamide and TPAE, especially derived from the whole aliphatic moieties, are flammable materials and show a low vertical combustion rating. Both aliphatic polyamide and TPAE are reported with a vertical combustion rating below V–2 [50,51], which is far from the flame-retardancy requirement of electronic, electrical, and automotive applications. The digital photographs of the samples after combustion are shown in Figure 5a, including TPAE-22, PA1212, and PA1212/Si12 copolymers. The difference of the combustion state among the samples is apparent. Under the same conditions, PA1212/Si12 presents a much lower burning rate, especially when the content of the Si12 unit is higher than 10 mol% (PA1212/Si12-20), while severe burning occurred in TPAE-22 and PA1212. Moreover, the presence of the BATS unit strongly suppresses the dripping phenomenon. Conspicuous melt dripping can be observed only in the PA1212/Si12-5 sample. When compared with TPAE-22 and PA1212, the dripping phenomenon in PA1212/Si12-5 sample is much less severe. Once the content of Si12 unit is raised to 10 mol% and higher, melt dripping becomes difficult to identify during the combustion. Although the dripping process tends to remove the heat to delay the combustion, the melt dripping, especially flammable dripping, is considered a second threat of ignition. Therefore, the anti-ignition performance of PA1212/Si12 copolymers is distinctly improved compared with TPAE-22 and PA1212. According to its UL-94 rating, the new series is upgraded to V–1 level. It should be noted that, even for PA1212/Si12-40, the content of Si atoms is still at a very low level, 4 wt.%, which should be the reason the copolymer did not pass the V–0 rating test. In addition, the upgraded flame-retardation level of the new series will facilitate its modification process and application.

Heat release rate (HRR) and total heat release (THR) are quite consistent with the vertical combustion rating result. The curves of PA1212/Si12 copolymers are presented in Figure 5b,c and the details are listed in Table 4. The peak temperature (*T*_p_) in HRR test of the new series shifts to a lower value with an increasing content of the Si12 unit, accompanied by a declining of the peak of heat release rate (PHRR). Specifically, *T*_p_ and PHRR are modulated from 482 °C to 473 °C and 1348 W·g^−1^ to 827 W·g^−1^, respectively, from PA1212/Si12-5 to PA1212/Si12-40. Generally, lower PHRR indicates better fire safety. In addition, similar to the variation tendency of PHRR, THR decreases from 34.5 kJ·g^−1^ of PA1212/Si12-5 to 31.2 kJ·g^−1^ of PA1212/Si12-40. In general, the HRR and THR results indicate better fire safety of the new series, which is regulated by the Si12 unit-content.

Together with the TG results under air atmosphere and FT-IR of the residue, we can speculate the anti-ignition process of the new series as follows. First, the silicone moieties in the copolymers tend to migrate to the surface of the material spontaneously due to the low surface energy of silicon atoms [52]. In addition, the migrated silicone moieties will generate a Si-C dense barrier layer to delay the combustion [36]. After ignition, the Si-C dense barrier layer immediately decomposes into SiO_2_ layer, illustrated by the white coating observed on the surface of the burned samples in Figure 5a. Following this, the SiO_2_ layer will extinguish the flame by rapidly isolating the sample from the oxygen. The spontaneous migration of the silicon moiety and the formation of the SiO_2_ isolation layer synergistically improves the flame retardancy of PA1212/Si12 copolymers.

In addition, it can be observed that the new self-flame retardant polyamide elastomer is white and translucent, similar in appearance to TPAE-22 and PA1212. This feature will favor the expansion of the application of TPAE in scenarios with rigid requirements for fire safety and material appearance, since darkening of the color is inevitable in the use of a phosphorus component as the solution to design self-extinguishing TPAE or polyamide [53,54].

### 3.6. Surface Properties and Anti-Fouling Test

**Figure 6:**
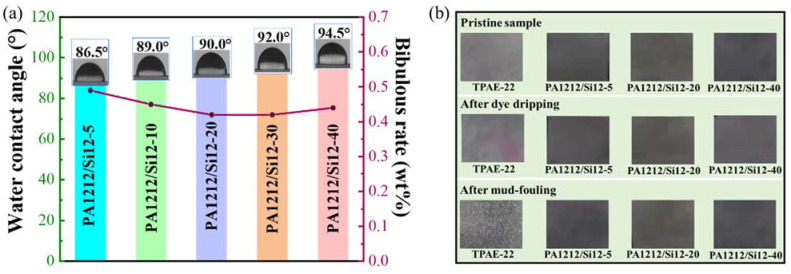
(**a**) Water contact angle of PA1212/Si12 copolymers; (**b**) The digital images for copolymers and TPAE-22 after dye dripping and mud contamination tests.

Due to the high affinity between the amide group and H_2_O, water absorption presents an issue of significant concern regarding the application of polyamides, especially in precision manufacturing. In addition, the commercial TPAE, poly (ether-*b*-amide), exhibits an even higher water absorption rate. Although it bears satisfactory application as a permanent antistatic additive, the dimensional and environmental stability of this form of TPAE presents a cause for great concern when used as a basic resin for manufacturing parts. As shown in Figure 6a and Table S4, the bibulous rates of the new copolymers were determined. PA1212/Si12 copolymers present much lower values than PA1212 and TPAE-22. The 24-h saturated water absorption rates of PA1212/Si12 copolymers are maintained around 0.45%, which is 30–40% lower than that of PA1212, and 60–70% lower than that of TPAE-22. Thus, much better dimensional stability, especially in moist conditions, of the new series can be expected, which will facilitate its application as a basic resin for manufacturing.

The water contact angles on the copolymer films were also measured. With the increasing Si12 unit content, the angle increases from 86.5° of PA1212/Si12-5 to 95° of PA1212/Si12-40, while the contact angles of PA1212 and TPAE-22 are 81° and 31°, respectively. The large gap in the water contact angle accounts for the highly decreased bibulous rates, especially compared with TPAE-22. Understandably, the low surface energy nature of siloxane moiety regulates the surface energy of PA1212/Si12 copolymers. The higher the Si12 unit content, the lower the surface energy the copolymer possesses, and the higher the contact angle will be. Finally, the character of the copolyamide successfully changes from hydrophilic to hydrophobic, which indicates that the series may show more stable performance in complex environments.

Anti-fouling properties of the film specimens with a 0.5 mm thickness were further evaluated to illustrate the benefit of the surface property change of PA1212/Si12 copolymers. Figure 6b and Figure S8 show the digital images of copolymers after dye dripping and mud contamination tests. It is evident that all PA1212/Si12 specimens remain essentially unchanged. The specimens remain as clean and clear as they were before the test. However, the performance of TPAE-22 is completely different. The distinct red traces are left on TPAE-22 films after dye dripping for 10 s. Although the surface of the specimen can be cleaned after mud contamination, numerous white spots appear in TPAE-22 films. This indicates that the water absorbed during the fouling process induces changes in TPAE-22 matrix, which may be caused by the phase separation. Consequently, this will have a destructive impact on the performance of the matrix, and lead to a sharp reduction in product service life. Benefitted by the excellent water repellency brought about by the BATS moiety, PA1212/Si12 copolymers display outstanding environmental stability, which is crucial for long-term applications.

### 3.7. Fluorescence Properties

**Figure 7:**
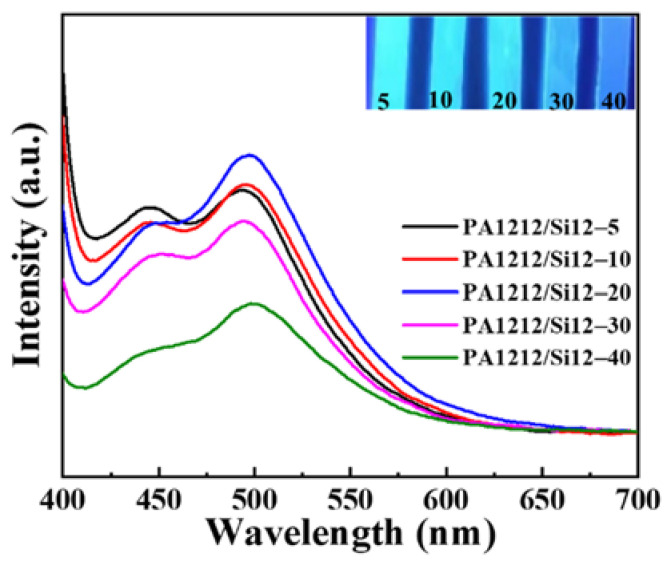
Fluorescence–emission curves of PA1212/Si12 copolymers.

Recently, non-conventional luminescent polymers (NLPs), which possess no remarkable conjugates but electro-rich moieties as chromophores, have attracted considerable interest, due to their promising applications in optoelectronic, biological and medical fields. Excitingly, a conspicuous fluorescence emission phenomenon was observed in all PA1212/Si12 specimens. The copolymers emit bright blue fluorescence when exposed to ultraviolet (365 nm) light source, as shown in Figure 7. As the amide-group-containing polymers [55,56,57] and the organosilicons [58,59] have been reported to exhibit non-conventional fluorescence, it is reasonable to attribute the luminescence of PA1212/Si12 copolymers to the synergistic contribution of the amide moieties and siloxane moieties. Although the exploration of the luminescence mechanism remains in its infant stages, through space conjunction (TSC) among the non-conventional chromophores induced by cluster formation is the most acceptable one. In addition, the phenomena are rationalized by the clustering-triggered emission (CTE) [60]. Hydrogen bonding is a common but important interaction that promotes the formation of the clusters [61,62,63,64], in polyamides and organosilicons. Furthermore, N-Si coordination bonding and improved aggregation efficiency facilitated by the high flexibility of the organosilicon also account for the silicon fluorescence emission. All the aforementioned factors result in TSC in the copolymer followed by non-conventional luminescence. More interestingly, the brightness of the emission light decreases with increases in Si12 unit content, corresponding to the intensity decline of the emission from PA1212/Si12-5 to PA1212/Si12-40 in the fluorescence spectra. As discussed above, with the increasing content of the Si12 unit, both the *T*_g_ and *X*_c_ decrease, which implies the constant decline of the interactions in both amorphous and crystalline phases. Thus, resulting cluster-triggered fluorescence emission is found to decrease.

## 4. Conclusions

In summary, a series of PA1212/Si12 copolymers were effectively designed and synthesized via a facile one-pot polycondensation route firstly based on LA, DMDA and BATS monomers and the chemical structure was well confirmed by FTIR and ^1^H-NMR. These prepared copolymers exhibit robust mechanical properties tailored by the Si12 unit content. From PA1212/Si12-20, the copolymer transits from plastic to thermoplastic elastomers. The improved compatibility of the silicon phase and polyamide phase and the induced crystalline phase transition from α-form to γ-form may synergistically result in the elastic stress–strain behavior. Moreover, compared with the traditional TPAE, the introduced Si12 unit with a higher bond energy and lower surface energy endows the PA1212/Si12 copolymers with excellent thermal stability and humidity tolerance. Furthermore, the novel series copolymer exhibits self-extinguishing and anti-fouling properties. Interestingly, PA1212/Si12 copolymers also exhibit blue luminescence when excited by a 365 nm light source. Owing to the facile synthesis route, robust mechanical properties, high-level fire safety and multifunctional properties, this silicone-containing polyamide elastomer will further broaden the application potential of TPAE, such as in electronic devices and the auto industry.

## Figures and Tables

**Table 1:** The parameters of the copolymers.

Sample	Content of Si12 Unit (mol%)		*M*_n_^b^ (kg/mol)	*M*_w_^b^ (kg/mol)	PDI ^c^	[*ƞ*] ^c^ (dL/g)	Residual Rate of Monomer (wt%) ^d^
	Theoretical Value	Calculated Value ^a^					
PA1212/Si12–5	5	3.2	6.8	17	2.4	1.2	1.6
PA1212/Si12–10	10	6.3	8.2	19	2.3	1.4	2.2
PA1212/Si12–20	20	13.9	7.3	16	2.2	1.3	2.9
PA1212/Si12–30	30	20.4	8.2	20	2.4	1.8	2.2
PA1212/Si12–40	40	26.1	5.5	13	1.9	1.9	3.2

^a^ Calculated from ^1^H NMR; ^b^ Determined by GPC; ^c^ Determined by Ubbelohde viscometer; ^d^ Determined by Soxhlet apparatus.

**Table 3:** The results of PA1212/Si12 copolymers from TGA.

Sample	N_2_ Atmosphere				Air Atmosphere			
	*T*_5%_ (°C)	*T*_max1_ (°C)	*T*_max2_ (°C)	Residue (%)	*T*_5%_ (°C)	*T*_max1_ (°C)	*T*_max2_ (°C)	Residue (%)
PA1212/Si12–5	430	463	559	1.49	411	432	529	2.21
PA1212/Si12–10	435	463	560	1.89	390	451	537	2.56
PA1212/Si12–20	423	464	556	1.88	362	430	546	3.10
PA1212/Si12–30	427	469	549	1.50	365	427	543	4.55
PA1212/Si12–40	425	473	547	1.75	375	438	546	4.75

**Table 4:** Combustion performance of PA1212/Si12 copolymers.

Sample	*T*_p_ (°C)	PHRR (W/g)	THR (kJ/g)	UL–94 Rating
PA1212/Si12–5	482	1348	34.5	V–2
PA1212/Si12–10	464	1142	33.0	V–2
PA1212/Si12–20	474	1064	31.8	V–1
PA1212/Si12–30	474	1025	33.6	V–1
PA1212/Si12–40	473	827	31.5	V–1

## Data Availability

Data available on request from the corresponding author.

## Supplementary Materials

The following supporting information can be downloaded at: https://www.mdpi.com/article/10.3390/polym14091919/s1, Figure S1: FT-IR and ^1^H NMR spectra of BATS, LA and DMDA monomers; Figure S2: Determination of *T*_g_ of PA1212/Si12 copolymers by DSC; Figure S3: Determination of Δ*H* of PA1212/Si12 copolymers by DSC; Figure S4: The WAXD pattern of PA1212; Figure S5: Deconvolution of Amide I band in IR spectra of PA1212/Si12 copolymers; Figure S6: TGA and DTG curves of PA1212/Si12 copolymers; Figure S7: FT-IR spectra of PA1212/Si12-40 after combustion; Figure S8: The digital images for PA1212/Si12-10 and -30 after dye dripping and mud contamination test; Table S1: The DSC test results of PA1212; Table S2: The deconvolution results and HBI of PA1212/Si12 copolymers; Table S3: TG characteristic parameters of PA1212 and TPAE-22; Table S4: Water contact angle and bibulous rate of PA1212 and TPAE-22.
