# Supplementary material for: Inherent Flame-Retardant, Humid Environment Stable and Blue Luminescent Polyamide Elastomer Regulated by Siloxane Moiety
Source: Polymers (Basel). 2022 May 9;14(9):1919. doi: 10.3390/polym14091919 (PMC9104639; doi:10.3390/polym14091919)
Supplement: Supplementary file 1 [file polymers-14-01919-s001.zip › polymers-1697737-supplementary.pdf]

polymers

MDPI

Supporting Information

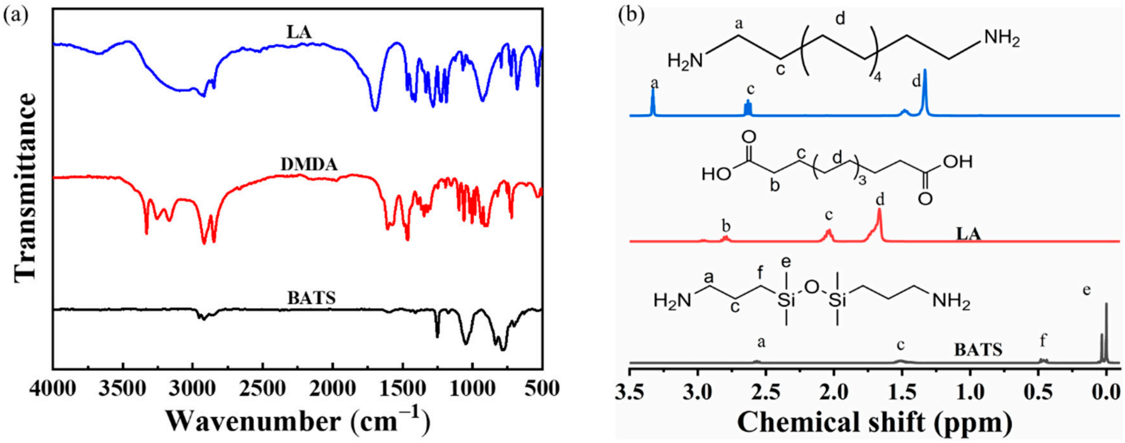

Figure S1. FT-IR (**a**) and ^{1}H NMR (**b**) spectra of BATS, LA and DMDA monomers.

Table S1. The DSC test results of PA1212.

Sample	T_g (°C)	T_m (°C)	T_c (°C)	ΔT (°C)	ΔH (J/g)	X_c[b] (%)
PA1212[a]	43	182	161	26	58.6	48.5

[a] homemade and [η]= 2.2 dL/g; [b] the standard enthalpy is 121.8 J/g[43].

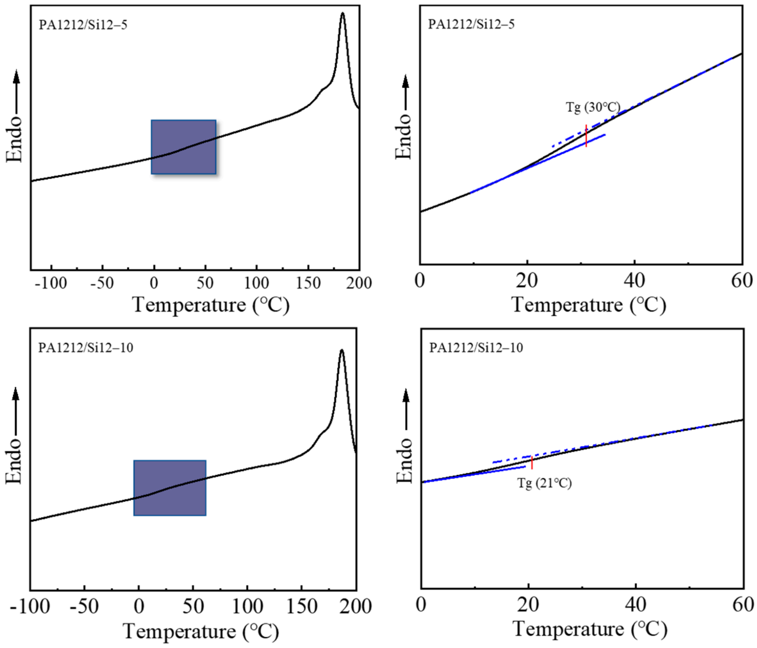

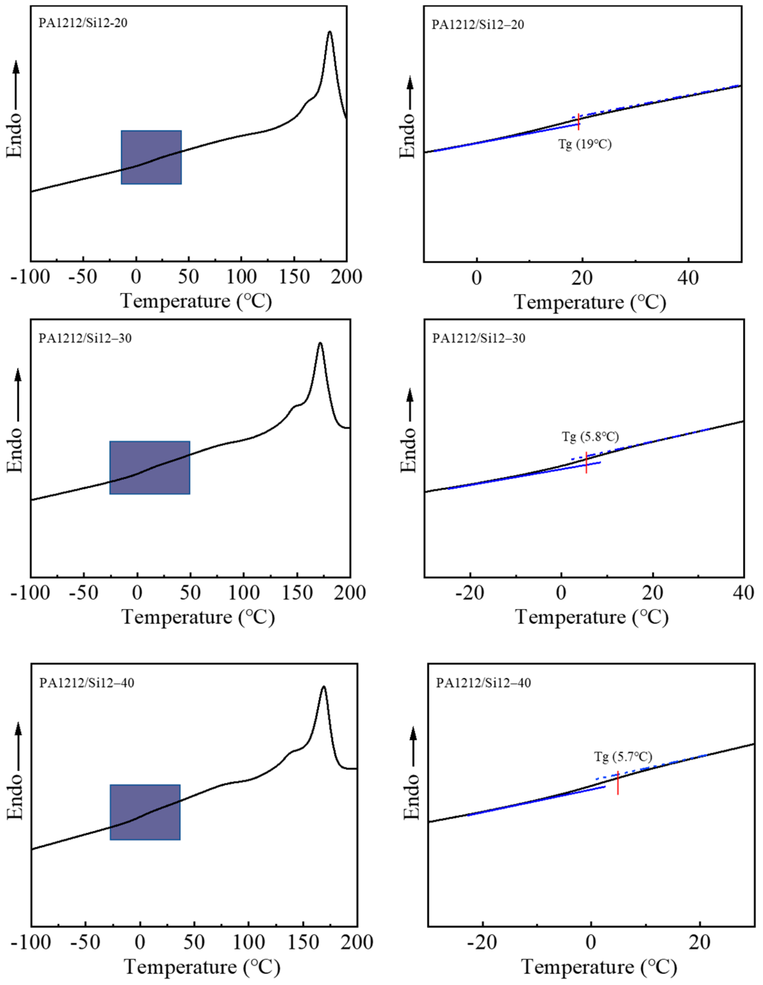

Figure S2. Determination of T_g of PA1212/Si12 copolymers by DSC (the second heating curves, 100 °C/min, under N_2 atmosphere).

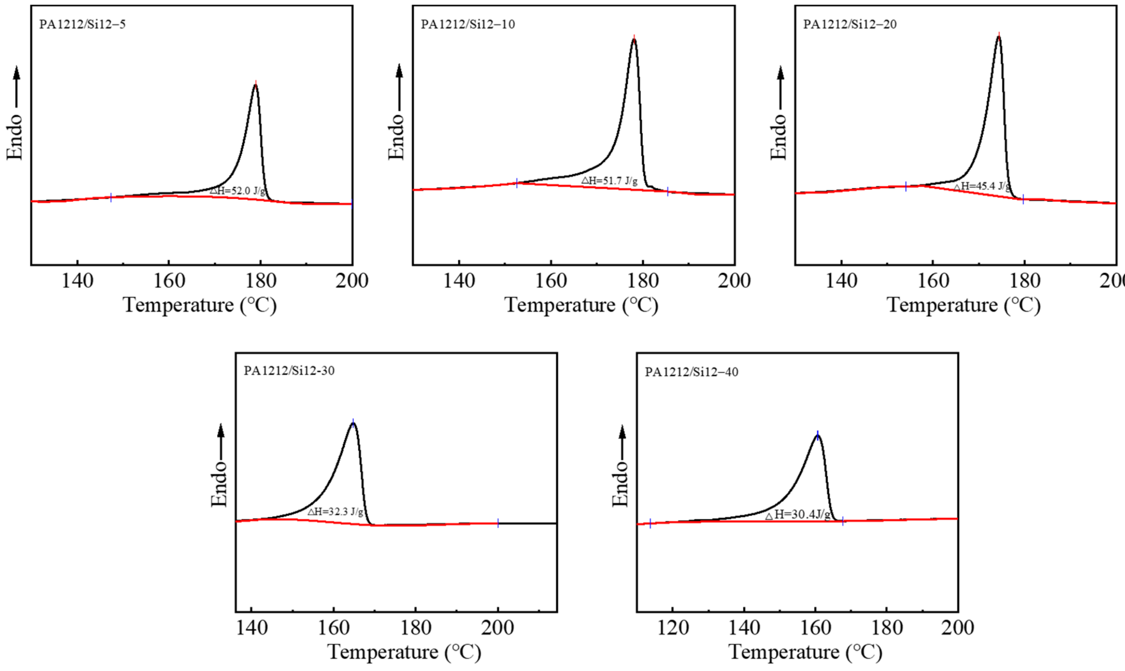

Figure S3. Determination of Δ*H* of PA1212/Si12 copolymers by DSC (the second heating curves, 10 °C/min, under N_2 atmosphere).

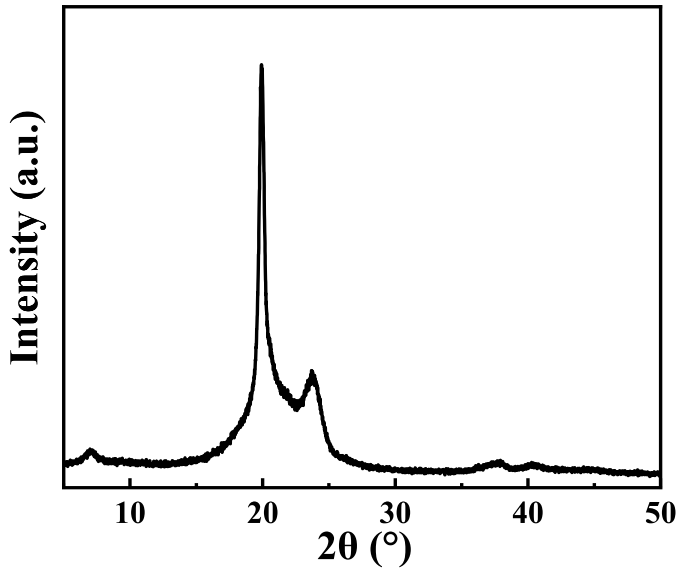

Figure S4. The WAXD pattern of PA1212.

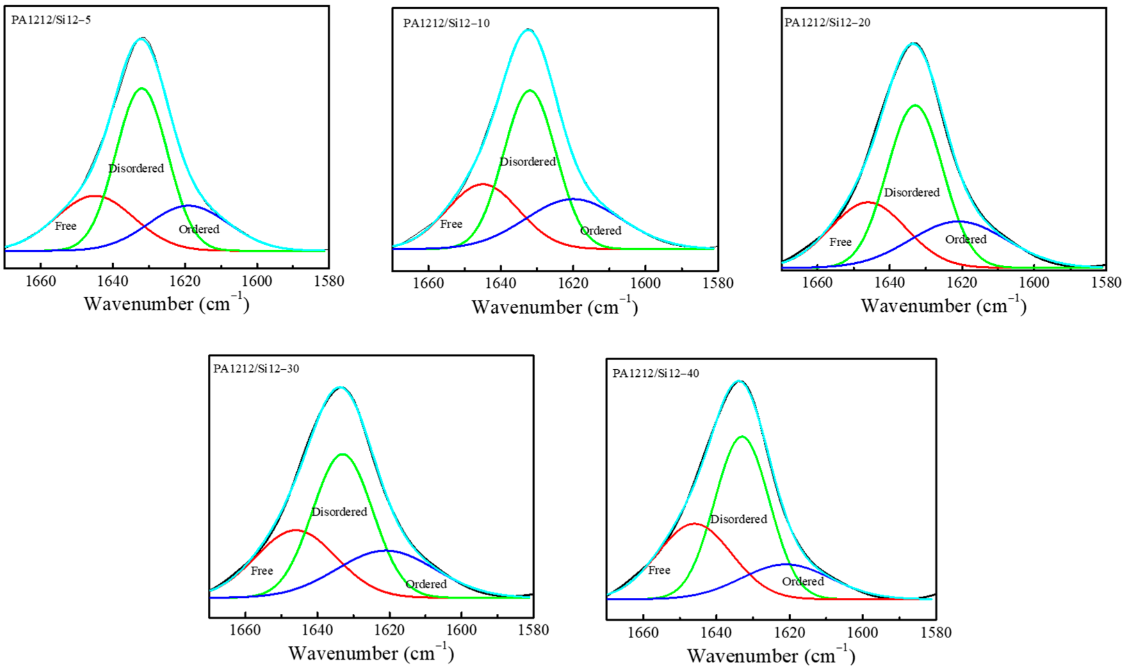

Figure S5. Deconvolution of Amide I band in IR spectra of PA1212/Si12 copolymers.

Table S2. The deconvolution results and HBI of PA1212/Si12 copolymers.

Sample	Peak Area of Free C=O	Peak Area of disor-dered- hydrogen-bonded C=O	Peak Area of ordered-hydrogen-bonded C=O	HBI
PA1212/Si12–5	1.27	2.50	1.07	2.79
PA1212/Si12–10	1.10	1.97	1.13	2.81
PA1212/Si12–20	1.71	3.12	1.53	2.58
PA1212/Si12–30	1.62	2.58	1.39	2.45
PA1212/Si12–40	1.39	2.21	0.77	2.14

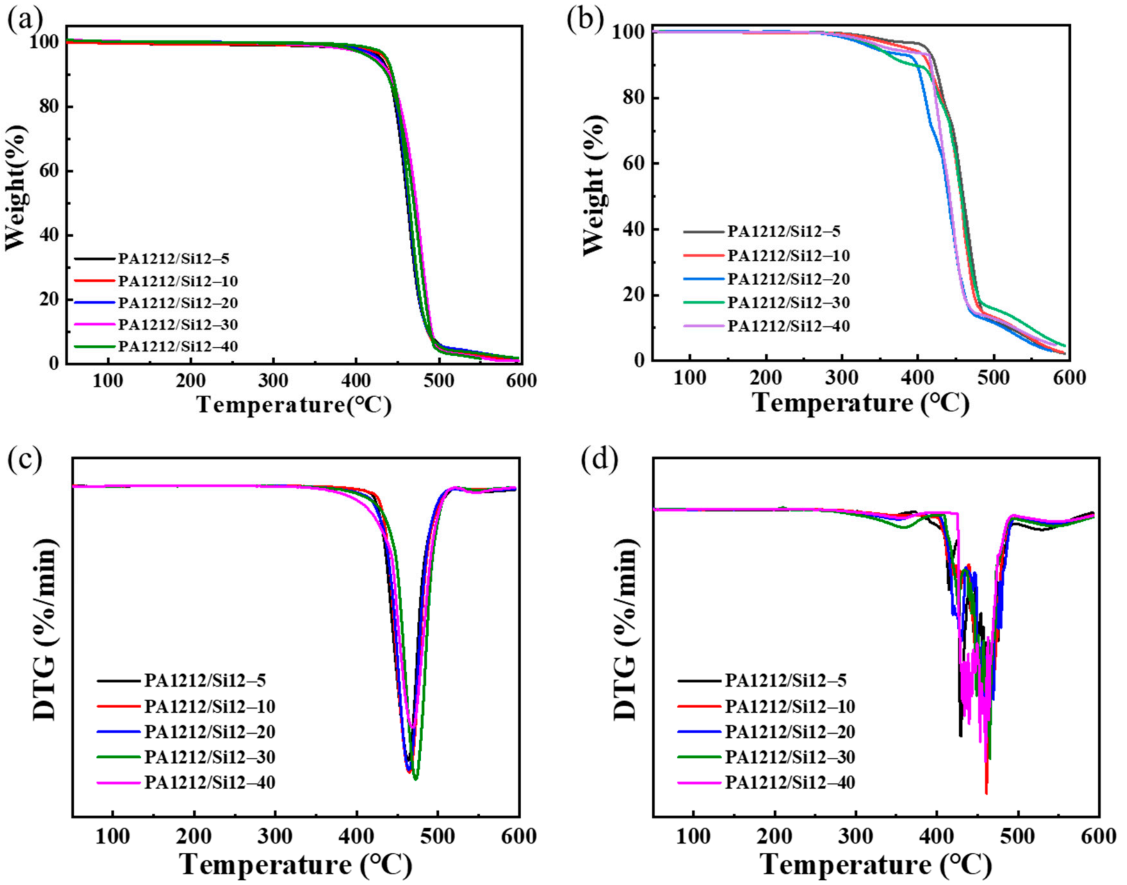

Figure S6. TGA and DTG curves of PA1212/Si12 copolymers (**a**) and (**c**) N_2 atmosphere; (**b**) and (**d**) under air atmosphere.

Table S3. TG characteristic parameters of PA1212 and TPAE-22.

Sample	N_2 atmosphere			Air atmosphere			
	$T_{5\%}$ (°C)	T_{max1} (°C)	Residue (%)	$T_{5\%}$ (°C)	T_{max1} (°C)	T_{max2} (°C)	Residue (%)
PA1212[a]	405	455	1.46	367	455	532	0
TPAE−22[b]	392	434	0.26	325	409	530	1.67

[a] [*η*] of PA1212 is 2.2 dL/g; [b] TPAE−22 was synthesized by PA1212 prepolymer and ED2003 (purchased from Huntsman, USA)[4] and [*η*] is 1.7 dL/g.

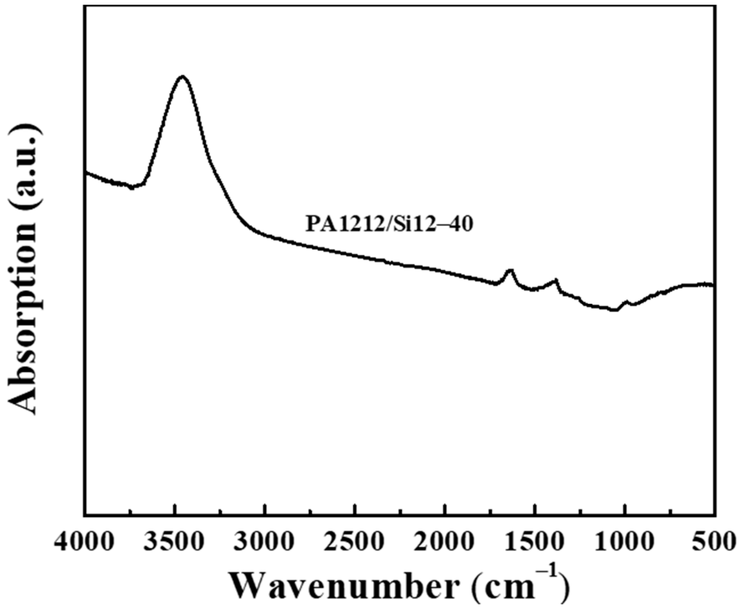

Figure S7. FT−IR spectra of PA1212/Si12−40 after combustion.

Table S4. Water contact angle and bibulous rate of PA1212 and TPAE.

Sample	Water contact angle (°)	Bibulous rate (%)
PA1212	84	0.63
TPAE−22	31	1.39

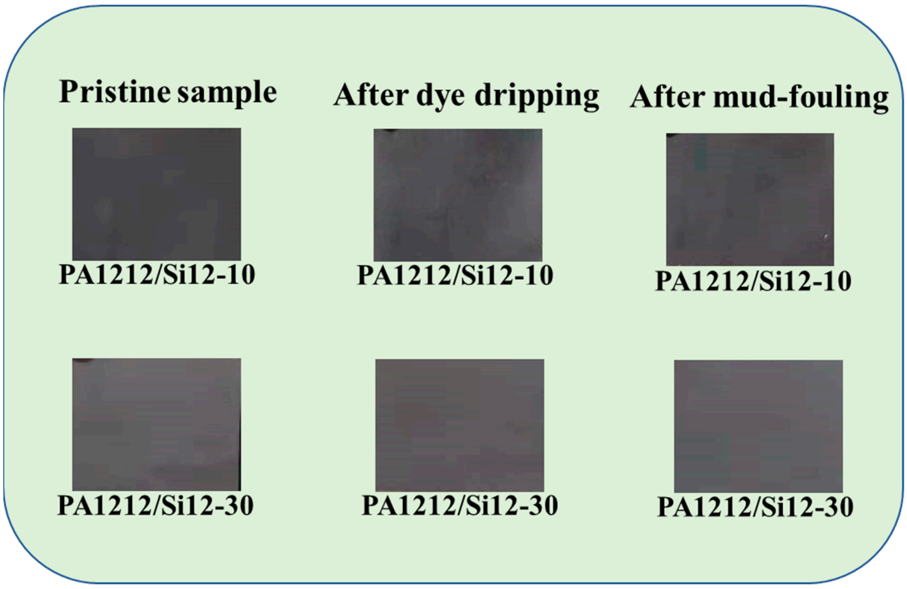

Figure S8. The digital images for PA1212/Si12−10 and −30 after dye dripping and mud contamination tests.
